# Scoping review of equity considerations in digitally enabled patient education interventions for self-managing chronic musculoskeletal pain

**DOI:** 10.3389/fpubh.2026.1954179

**Published:** 2026-09-30

**Authors:** Hina Khalid, Nourah Basalem, Sanniya Saleem, Hurmah Shoaib, Nida Ahmed, Kanwal Baloch, Ali Hindi, Sabine N. van der Veer, Syed Mustafa Ali

**Affiliations:** 1 BMY Health, Lahore, Pakistan; 2 Centre for Health Informatics, Division of Informatics, Imaging and Data Science, Manchester Academic Health Science Centre, The University of Manchester, Manchester, United Kingdom; 3 College of Applied Medical Sciences, Prince Sattam Bin Abdulaziz University, Al-Kharj, Saudi Arabia; 4 Royal Bournemouth Hospital, University Hospitals Dorset NHS Foundation Trust, Bournemouth, United Kingdom; 5 Karachi Institute of Medical Sciences, Karachi, Pakistan; 6 Division of Pharmacy and Optometry, School of Health Sciences, Faculty of Biology, Medicine and Health, The University of Manchester, Manchester, United Kingdom; 7 National Institute for Health and Care Research, Applied Research Collaboration – Greater Manchester, Manchester, United Kingdom

**Keywords:** chronic musculoskeletal pain, digital health, health equity, patient education, self-efficacy, self-management

## Abstract

**Background and objectives:**

Patient education is critically important for supporting self-management of chronic musculoskeletal pain, but it is unclear if and how existing tools or interventions are meeting diverse pain management needs. Therefore, this review aimed to identify and characterise digitally enabled pain education tools or interventions and explore the extent to which they meet diverse needs.

**Methods:**

We searched five electronic databases, including MEDLINE (via Ovid; Annexure A), Embase (via Ovid), CINAHL, PsycInfo and Google Scholar in January 2025, and screened articles based on pre-defined eligibility criteria. Articles were eligible if they reported on the development, evaluation and use of digitally enabled pain education tools or interventions, supporting pain self-management. Data was extracted and analysed descriptively.

**Results:**

Our search strategy yielded 12,545 search results, out of which 100 studies were included. Majority of these studies were published in North America and Europe (*n* = 86; 86%) and lacked focus on population subgroups having higher pain management needs (*n* = 86; 86%). Out of 39 named or unique interventions, 13 tools or interventions were tailored to meet diverse needs. Most of the studies (*n* = 81; 81%) reported the results of real-world testing and effectiveness evaluation. There were only few studies reporting on the early development stages (i.e., co-design studies and prototype testing). For effectiveness studies, pain intensity, pain interference, pain-related knowledge, self-efficacy, function, opioid use and quality of life were commonly measured patient-reported outcomes.

**Conclusion:**

Although there are several digitally enabled patient education tools or interventions to support pain self-management, fewer are available to address the diverse needs. Therefore, future research and development should focus on understanding and addressing these unmet needs for developing more inclusive and equitable digitally enabled patient education interventions.

## Introduction

Chronic musculoskeletal (MSK) pain is a widespread condition with significant personal, societal and economic burdens, including high healthcare costs, loss of productivity, and reduced quality of life (1–3). Globally, significant proportion of the population experiences chronic MSK pain, with approximately one in five individuals in the United States of America, Europe, Australia and up to one in three individuals in Asia (e.g., Cambodia) experience it (4, 5), but it is often inadequately managed (6). However, certain population groups, such as older adults, women, ethnic minorities and groups with low socioeconomic status are disproportionately affected by chronic MSK pain (7).

In addition to differences in chronic MSK pain prevalence and its outcomes, there are also differences in pain management needs, which are referred to as health equity considerations and recognised in the biopsychosocial model of pain management (8). Lack of considerations to these diverse needs may leave many without adequate support, limiting their access to pain management services and resulting in poorer pain management outcomes (9). Moreover, effective chronic MSK pain management requires multidisciplinary approach (10) and considering a wide range of treatment options, which are aligned with patients’ personal preferences and their unique circumstances (9, 11). Recognising these unique characteristics may lead to recognising the social context in which people live and manage their pain conditions. This highlights the need for managing chronic MSK pain in a broader context (12), need for which is further highlighted because of infrequent clinical consultations and discordance between patients and pain clinicians (13, 14).

Pain self-management is crucial for effective pain management. It reflects deliberate efforts by a person living with chronic MSK pain to manage it, its causes and effects on physical and emotional wellbeing (15, 16), precursor of which is self-efficacy, i.e., one’s own ability to manage pain (17). Patient education is a key component of pain self-management, which is also supported by evidence and pain management guidelines (10, 18, 19). Patient education empowers individuals to participate in their treatment actively, reduces their reliance on clinical interventions (20, 21) and motivates them to change behaviours (22, 23) by addressing the biopsychosocial aspects of pain (24). However, patient education as a critical care element, is underutilised and inadequately evaluated (25).

The increasing demand for digitally enabled patient education tools highlights their potential to transform chronic MSK pain management by addressing limited access to specialised pain management services (26), and enhancing health education by delivering tailored and engaging content (27, 28). While these tools offer significant benefits, challenges such as accessibility for older or less tech-savvy users persist. Moreover, existing reviews have looked at enablers and barriers without focusing on specific patient characteristics to identify their educational needs and how these are being met (29, 30), particularly for those who have higher pain management needs (referred as priority groups) (27, 31, 32). Therefore, this scoping review aimed to identify and characterise digitally enabled pain education tools or interventions and target populations for whom these were developed or used. Specific objectives of this review were:

(a) Identify and characterise studies focusing on digitally enabled patient education as (part of) a pain self-management strategy;
(b) Explore to what extent digitally enabled patient education tools or interventions have been co-designed and evaluated;
(c) Characterise features of digital-enabled patient education tools or interventions (e.g., content, delivery mode) and to what extent they are equitable.

## Methods

We conducted and reported a scoping review in accordance with the guidelines of the PRISMA-ScR (Preferred Reporting Items for Systematic Reviews and Meta-Analyses Extension for Scoping Reviews) (33). We conducted this scoping review to explore existing literature and identify the type of evidence available, without the need for its rigorous quality appraisal, which is typically required in systematic reviews (33, 34). We did not register our review protocol, though we searched key databases of systematic review protocols to avoid duplication of efforts.

### Inclusion and exclusion criteria

We used the population, concept, and context (PCC) framework to formulate our review question and described inclusion and exclusion criteria. We included empirical studies, including quantitative, qualitative, and mixed methods research with full text published in the English language after 1990 (35), when pain education was first acknowledged as a requirement for pain management. We also included case series with at least 10 cases. Eligibility criteria for the components of the PCC framework are outlined below:

*Population*: The target population is adults (aged 16 years or older) living with musculoskeletal chronic pain conditions, including chronic primary MSK pain (e.g., pain without any underlying medical condition), and chronic secondary MSK pain (e.g., pain caused by a musculoskeletal condition, such as osteoarthritis), or both. Population groups having underlying comorbid conditions such as diabetes, hypertension, regardless of their socio-demographic backgrounds were also included. In addition to adults living with chronic MSK pain as an intended target population, we also included studies involving other stakeholders as study populations, such as healthcare providers (e.g., physicians, nurses, physiotherapists) or other related experts (e.g., occupational therapists, dietitians/nutritionists, technology developers).

We excluded paediatric pain and pain caused by trauma, injury or surgery. Studies reporting on the education of healthcare professionals were excluded.

*Concept*: The concept of interest for this review was digitally enabled patient education tools or interventions (including, but not limited to telehealth, mobile application, web-based interventions, artificial intelligence, etc.) intended to assist people living with chronic MSK pain in self-managing their pain. Patient education is a structured process tailored to meet patients’ needs in developing or maintaining self-management skills and adopting or modifying behaviours helping patient to manage chronic MSK pain. This may involve: (a) delivering a structured education content to improve patient’s knowledge about chronic MSK pain; (b) collaborative decision making based on a shared understanding of the patient’s needs through goal-setting and action planning; and (c) and imparting practical skills training for behaviour change and maintenance (36). Example of digitally enabled patient education for self-managing pain is a chatbot (SELMA) (37), which is tailored based on user-reported pain intensity and duration, offered psychoeducation (i.e., explaining pain’s biopsychosocial nature) and CBT-based coping strategy modules (e.g., to support activity pacing, and managing fear of pain). To describe the characteristics of interventions, we mapped the included studies against intervention names (where provided by the authors, e.g., selfBack), rather than by their individual components (e.g., cognitive behavioural therapy).

Studies reporting on patient reported outcomes such as pain intensity, physical function, self-efficacy, which were directly associated with patient education tool or intervention were included. Studies were excluded if digital tools were not specifically intended for the patient education or pain self-management and rather used only for communication between healthcare professionals and patients.

*Context*: There were no restrictions on the context of this review. We considered home, community, workplace, clinical settings and country as suitable contexts. Studies conducted in the settings of acute care or emergencies were excluded because their management primarily focuses on immediate assessment and treatment, rather than the ongoing education and supported self-management required for chronic MSK pain.

### Search strategy

We searched five electronic databases in January 2025 by developing a comprehensive search strategy in consultation with a librarian and adopted search strings for each key search term, including patient education and chronic pain and adapted them for each electronic database. Instead of using chronic MSK pain, we used ‘chronic pain’ as a search term to maximise search sensitivity. We searched MEDLINE (via Ovid; Annexure A), Embase (via Ovid), CINAHL, PsycInfo and Google Scholar. We searched for the first 80 pages of Google Scholar to retrieve optimal search results (38).

### Screening process

Following the searches and after deduplication (using duplication function in Microsoft Excel), we conducted pilot screening on first 100 titles, discussed pilot screening results, and refined eligibility criteria. After that, six reviewers in three pairs completed the screening of titles and abstracts in duplicates, discussed and resolved disagreements within each pair through consensus, with any disagreement resolved through discussion with a third reviewer in weekly meetings (SNvdV or SMA). The same process was followed for full-text screening and extracting data from eligible studies. Each pair screened one third of all articles. We used Microsoft Excel to manage search and screening and extraction results.

### Data charting

Six reviewers extracted data from eligible studies by using a data charting template. We extracted data related to characteristics of included studies, study population and interventions developed or tested. For data extraction related to priority subgroups, we used PROGRESS plus framework (39) to guide identification of diverse subgroups and for translational stages (see Table 1) we took guidance from previous reviews (40, 41).

**Table 1:** Stages of the translational pathway and their description.

Translational stage	Description
Co-design or user requirement	Studies focusing on collection of requirements regarding digitally enabled patient education to support pain self-management
Prototype testing	Studies presenting initial prototypes of digitally enabled patient education tools or interventions to take feedback in controlled settings, such as lab, university or community venue.
Real world testing	Studies focusing on evaluating user engagement with tools and testing hypothesis in real-world settings, such as homes
Evaluation for effectiveness	Studies evaluating the effectiveness of interventions through robust study designs, such as RCTs, quasi-experimental.
Post effectiveness qualitative evaluation	Studies focus on gathering feedback to explore approaches to further optimise the results of real-world testing and intervention effectiveness.

We also extracted data regarding patient reported outcomes which were measured for demonstrating intervention’s effectiveness and participants’ experience. The template was piloted and refined before extracting data from all eligible studies. Data extraction of one quarter of the eligible studies was done in duplicate for quality assurance, and results were discussed in pairs of two and with the third reviewer to address any disagreement (SMA). Any further consensus needed was discussed in group discussions to develop a shared understanding of extraction items and to ensure consistency in data extraction. We extracted data and analysed it against each of the three objectives and summarised descriptively in frequencies and percentages.

## Results

Search of five databases yielded 12,545 search results, from which 1,148 duplicates were removed. After deduplication, 11,397 articles were screened, out of which 100 articles were considered eligible and included in this review (Figure 1).

**Figure 1:**
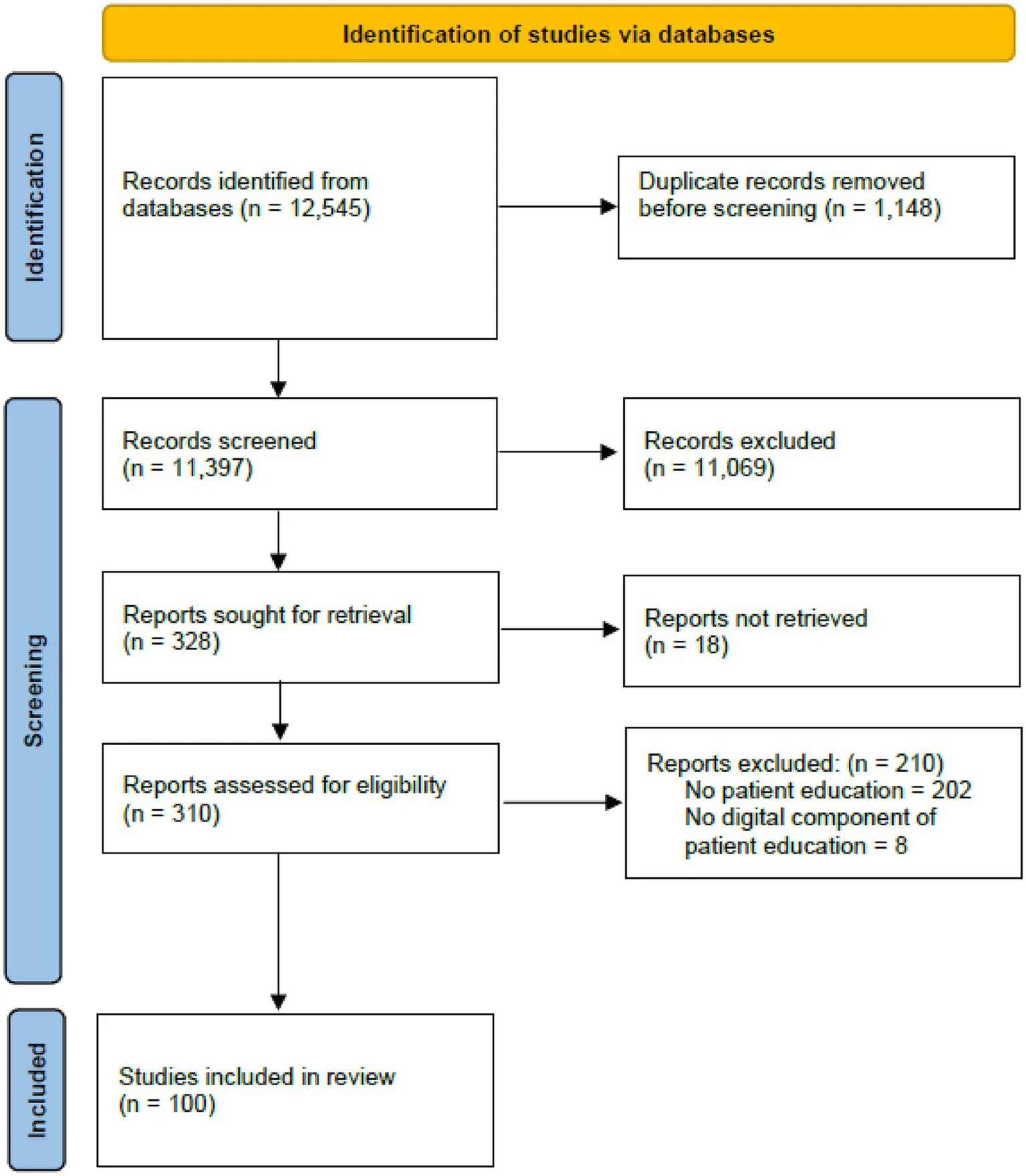
PRISMA flow diagram.

### Study characteristics

The scoping review included 100 articles, majority of which were published in North America and Europe (*n* = 86; 86%) and recruited people with primary chronic MSK pain (*n* = 45; 45%) from clinical settings (*n* = 54; 54%). Majority of the studies lacked focus on priority subgroups (*n* = 86; 86%). Among the determinants describing priority populations subgroups, age was the most common determinant (*n* = 7), followed by place of residence (*n* = 4), ethnicity (*n* = 3), gender (*n* = 1) and education level (*n* = 1). Detailed study characteristics are presented in Table 2 and complete list of included studies is included as a Supplementary material – Annexure B.

**Table 2:** Study characteristics (*n* = 100).

Variables	Number (percentage)
Geographical location	
North America	44 (44)
Europe	41 (41)
South America	1 (1)
Australia	7 (7)
Asia	7 (7)
Study settings	
Clinical	54 (54)
Community	35 (35)
Both clinical and community	9 (9)
University or lab	2 (2)
Study population type	
People with pain/painful condition	98 (98)
People with pain and healthcare professionals	2 (2)
MSK Pain type	
Primary pain	45 (45)
Secondary pain	36 (36)
Both	11 (11)
Unclear	8 (8)
Study population size	
<50	32 (32)
50–100	25 (25)
101–300	26 (26)
>300	17 (17)
Priority population groups	
Yes*	14 (14)
No	86 (86)

*Studies considered at least one of the social determinants for participants’ recruitment and/or intervention development or evaluation, such as age, location, gender, ethnicity, and disability.

#### State of development and evaluation of digitally enabled patient education tools or interventions

Majority of the studies (*n* = 92; 92%) reported on at least one of the five translational stages, as presented in Table 3. Among them, most of the studies (*n* = 81; 81%) reported the results of real-world testing and effectiveness evaluation, and fewer studies explored people’s experience of intervention (*n* = 7; 7%). Moreover, there were only few studies reporting on the early stages of their development (*n* = 4; 4%).

**Table 3:** Mapping of the eligible studies to translations stages.

Translational stage	Number of studies *N* (%)
Co-design or user requirement	1 (1)
Prototype testing	3 (3)
Real world testing	34 (34)
Evaluation for effectiveness	47 (47)
Post effectiveness qualitative evaluation	7 (7)
Multiple (>1 of the above stages)	8 (8)

This review found a wide variety of patient reported outcomes to evaluate feasibility and effectiveness of interventions. For the studies evaluating the effectiveness of digital interventions or tools, pain intensity, pain interference, pain-related knowledge, self-efficacy, function (both mental and physical), disability, opioid use and quality of life were more commonly measured patient reported outcomes. However, pain acceptance, fear avoidance, emotional response, patient empowerment, patient activation, patient satisfaction, health literacy, and well-being were less commonly measured patient reported outcomes to demonstrate intervention effectiveness. Moreover, in studies related to early stages of development, patient reported outcome measures were not related to pain. The commonly used patient reported outcomes were acceptance, feasibility and perceived usefulness of digital interventions.

#### Features of digitally enabled patient education tools or interventions

Among the 100 included studies, 56 studies mentioned a name of digital intervention or tool, reporting on 39 unique interventions.

##### Type of content and their mode of delivery

Out of 39 unique interventions, webpages were the most common method of delivering content (*n* = 23; 59%), which was in the form of text, audios, videos, infographics or illustrations. These webpages were often combined with synchronous components (e.g., interactive sessions) (27, 42–63).

Smartphone applications were the second most common delivery mode (n = 12; 31%), offering content that ranged from text-based materials to interactive formats, including virtual classes (64–75) and synchronised wearable devices (76). Less common methods were synchronous communication methods, such as telephone and group video calls (77–84) and asynchronous communication tools such as e-mails distributing text-based materials (85) and portable storage devices, e.g., DVDs or USB drives for delivering audio-visual education (86–89).

##### Components of patient education and their tailoring

Components of patient education varied widely across interventions, both in scope and in the degree of tailoring. Interventions delivered via text-heavy webpages often targeted knowledge acquisition (55), whereas those using multimedia content more frequently combined knowledge enhancement with behaviour change strategies (51). Synchronous communication methods such as telephone or video calls were primarily associated with knowledge-based educational interventions, with fewer explicitly aiming to modify behaviours (53). To facilitate behaviour change, the commonly adopted strategies were cognitive behavioural therapy (CBT), positive psychology intervention (PTI), acceptance and commitment therapy (ACT), mindfulness exercises and pain coping strategies.

Out of 39 unique interventions, 13 interventions incorporated at least one tailoring aspect based on language, pain assessment, physical and mental capabilities, literacy or personal goals. Out of 39 interventions, seven interventions were reported more than once (Table 4).

**Table 4:** Example digitally enabled patient education tools or interventions and their translational journey.

Name of tool or intervention	Period	Number of publications	Focused on priority population	Tailored to meet diverse needs
ONESELF	2007–2014	7	Yes	Yes ^1^
Goalistics chronic pain management program	2012–2023	5	No	Yes ^2^
painACTION	2010–2015	4	No	No
Prescription for education (RxEd)	2017	2	No	No
EPIO	2020–2025	2	No	No
Digital care program (DCP)	2022–2024	2	No	Yes ^3^
selfBACK	2023	2	No	Yes ^4^

^1^Language; Virtual library and gymnasium provided tailored multimedia contents to support learning and physical rehabilitation, also enabling chat with healthcare professional. ^2^Individualised custom plan and summary report based on the results of pain assessment. ^3^Intervention was adjusted patients’ condition and personal goals. ^4^Self-management recommendations were tailored to individual characteristics, symptoms, and progression by using the case-based reasoning methodology.

## Discussion

This scoping review included 100 studies describing a wide range of digitally enabled patient education tools or interventions for pain self-management, majority of which were published in North America and Europe. Participants were predominantly adults living with chronic MSK pain, while fewer studies had a focus on priority population subgroups (e.g., groups related to age, place of residence, ethnicity, gender and education level). Thirty-nine named and unique interventions were reported across 100 studies, out of which one third tool or interventions were tailored to diverse needs, for which personal goals and physical and mental capabilities were the common considerations. The digital content type varied significantly based on the interventions or tool’s primary objectives. While most interventions relied on text-based content delivered via webpages to improve knowledge, those utilising multimedia resources (e.g., audio-visual content) were designed to both improve knowledge and actively guide behaviour change. Majority of the studies reported on the effectiveness of interventions, commonly using pain intensity, function, disability, self-efficacy and quality of life as patient reported outcomes to demonstrate their effectiveness.

We found that the majority of the digitally enabled pain education interventions were developed and evaluated in developed countries with majority population groups. Fewer interventions reported on tailoring, with only seven studies considering language and literacy for people from different ethnic backgrounds and of lower literacy. However, evidence suggests that people from ethnic minority backgrounds living in developed countries have higher pain management needs and pose a greater burden on healthcare systems and societies (90–93), which the existing interventions are less likely to address. This suggests a lack of equity considerations during intervention development, which may result in widening of gaps between pain management needs and resources available to address them as also noted in other reviews related to chronic MSK pain management (40, 94, 95). Moreover, if interventions originally developed in high income or developed countries are equitable and inclusive, then their translation to developing countries may become economically viable with potential health benefits for poor or less developed nations. However, it is important to understand and identify differences between two wider contexts (96, 97), which should be the focus of future research and development agenda.

In this review, we have found that majority of the studies reported on delivering the content focused on improving pain-related knowledge and self-efficacy and ultimately informing behaviour change through setting personal goals and developing action plans. However, none of the content was related to dietary modifications, which are considered important for self-management of long-term conditions (98), including chronic MKS pain (99). The NICE guidelines on chronic pain, which were last updated in 2021, did not include any advice or recommendation on diet or nutrition (19). However, the Scottish Government has recently published guidance on the Quality Prescribing for Chronic Pain (2026–2029), which outlines the role of diet or nutrition in chronic MSK pain management (21). Moreover, none of studies or interventions explicitly mentioned lay or home-based remedies, which are increasingly being adopted for self-managing pain, particularly among people from culturally and linguistically diverse backgrounds (100, 101). This might be because of the lack of involvement of people with diverse backgrounds and lack of co-design studies with them.

This review has highlighted the underrepresentation of priority population subgroups in studies related to intervention development and evaluation, highlighting a potential for these groups to be adversely affected by interventions that lack tailoring. There are other reviews suggesting that digital interventions for pain management were either comparable or slightly better than usual care (51, 102, 103). Consistent with our review, these reviews also reported on studies conducted with majority populations, which might be the reason for comparable or slightly better outcomes owing to their lower pain management needs as compared to people from underserved, ethnically diverse and socioeconomically disadvantaged groups.

Focus on priority population subgroups, who would have additional pain management needs, was the strength of this review. However, there was a potential limitation in our search strategy review, i.e., our search lacked specificity and may have caused screening burden because we did not include search terms related to digital health. We decided not to include digital health related search terms because this field and related terminologies are evolving/changing rapidly and indexing issues are also reported (104). Moreover, our search strategy did not include searching commercially developed solutions, which are not reported in academic literature. Previous reviews suggest that digital health technologies reported in academic literature are different from publicly available digital health technologies, such as via app stores. Future reviews should focus on digital health solutions which are available to public via different routes to understand the current landscape of technologies supporting pain self-management. Moreover, researchers and pain management professionals should focus on involving people with higher pain management needs while developing and evaluating these interventions for greater positive health impact and wider societal benefits.

## Conclusion

There are several digitally enabled patient education interventions or tools to support pain self-management. Most of these interventions are developed and evaluated with majority population groups living in the developed countries. We found limited number of co-design studies and limited tailoring of interventions or tools highlighting the lack of equity considerations. This perpetuates the unmet pain management needs of the groups which are disproportionality affected with chronic MSK pain. Therefore, future research and development should focus on understanding and addressing these unmet needs for developing more inclusive and equitable digitally enabled patient education interventions, which would have positive health equity and societal impact.

## Data Availability

The original contributions presented in the study are included in the article/Supplementary material, further inquiries can be directed to the corresponding author/s.

## References

[1] Duenas M Ojeda B Salazar A Mico JA Failde I. A review of chronic pain impact on patients, their social environment and the health care system. J Pain Res. (2016) 9:457–67. doi: 10.2147/JPR.S105892, 27418853 PMC4935027

[2] Mann EG LeFort S VanDenKerkhof EG. Self-management interventions for chronic pain. Pain Manag. (2013) 3:211–22. doi: 10.2217/pmt.13.9, 24654764

[3] Zhu M Zhang J Liang D Qiu J Fu Y Zeng Z . Global and regional trends and projections of chronic pain from 1990 to 2035: analyses based on global burden of diseases study 2019. Br J Pain. (2025) 19:125–37. doi: 10.1177/20494637241310697, 39726775 PMC11669129

[4] Breivik H Collett B Ventafridda V Cohen R Gallacher D. Survey of chronic pain in Europe: prevalence, impact on daily life, and treatment. Eur J Pain. (2006) 10:287–7. doi: 10.1016/j.ejpain.2005.06.009, 16095934

[5] Mohamed Zaki LR Hairi NN. A systematic review of the prevalence and measurement of chronic pain in Asian adults. Pain Manag Nurs. (2015) 16:440–52. doi: 10.1016/j.pmn.2014.08.012, 25439125

[6] Wang J Doan LV. Clinical pain management: current practice and recent innovations in research. Cell Rep Med. (2024) 5:101786. doi: 10.1016/j.xcrm.2024.101786, 39383871 PMC11513809

[7] Mills SEE Nicolson KP Smith BH. Chronic pain: a review of its epidemiology and associated factors in population-based studies. Br J Anaesth. (2019) 123:e273–83. doi: 10.1016/j.bja.2019.03.023, 31079836 PMC6676152

[8] Gatchel RJ Peng YB Peters ML Fuchs PN Turk DC. The biopsychosocial approach to chronic pain: scientific advances and future directions. Psychol Bull. (2007) 133:581–624. doi: 10.1037/0033-2909.133.4.581, 17592957

[9] Cohen SP Wang EJ Roybal A Chen Y. Factors predicting outcomes from chronic pain management interventions. BMJ Med. (2025) 4:e001143. doi: 10.1136/bmjmed-2024-001143, 40735511 PMC12306485

[10] Faculty of Pain Medicine. (2021). *Core Standards for Pain Management Services in the UK - Second Edition*. Faculty of Pain Medicine.

[11] Ali SM Gambin A Chadwick H Dixon WG Crawford A Van der Veer SN. Strategies to optimise the health equity impact of digital pain self-reporting tools: a series of multi-stakeholder focus groups. Int J Equity Health. (2024) 23:233. doi: 10.1186/s12939-024-02299-w, 39529006 PMC11555918

[12] Patel P Green M Tram J Wang E Murphy M Abd-Elsayed A . Beyond the pain management clinic: the role of AI-integrated remote patient monitoring in chronic disease management – a narrative review. J Pain Res. (2024) 17:4223–37. doi: 10.2147/JPR.S494238, 39679431 PMC11646407

[13] Calpin P Imran A Harmon D. A comparison of expectations of physicians and patients with chronic pain for pain clinic visits. Pain Pract. (2017) 17:305–11. doi: 10.1111/papr.12428, 26992011

[14] Spink A Wagner I Orrock P. Common reported barriers and facilitators for self-management in adults with chronic musculoskeletal pain: a systematic review of qualitative studies. Musculoskeletal Sci Pract. (2021) 56:102433. doi: 10.1016/j.msksp.2021.102433, 34416557

[15] Kerns RD Burgess DJ Coleman BC Cook CE Farrokhi S Fritz JM . Self-Management of Chronic Pain: psychologically guided Core competencies for providers. Pain Med. (2022) 23:1815–9. doi: 10.1093/pm/pnac083, 35642906 PMC9629397

[16] McGillion M LeFort SM Webber K Stinson JN Lalloo C. "Pain Self-Management: Theory and Process for Clinicians". In: Clinical Pain Management. Hoboken, NJ: Wiley (2022). p. 261–71.

[17] Kawi J Yeh CH Grant L Thrul J Wu H Christo PJ . Adapting and evaluating a theory-driven, non-pharmacological intervention to self-manage pain. Healthcare (Basel). (2024) 12:969. doi: 10.3390/healthcare12100969, 38786380 PMC11121327

[18] Duhn P Wæhrens E Pedersen M Nielsen S Locht H Bliddal H . Effectiveness of patient education as a stand-alone intervention for patients with chronic widespread pain and fibromyalgia: a systematic review and meta-analysis of randomized trials. Scand J Rheumatol. (2023) 52:654–63. doi: 10.1080/03009742.2023.2192450, 37162478

[19] NICE. *Chronic Pain (Primary and Secondary) in Over 16s: Assessment of all Chronic Pain and Management of Chronic Primary Pain*. (2021). Available online at: https://www.nice.org.uk/guidance/ng193. 33939353

[20] Blake H Chaplin WJ Gupta A. The effectiveness of digital interventions for self-management of chronic pain in employment settings: a systematic review. Br Med Bull. (2024) 151:36–48. doi: 10.1093/bmb/ldae007, 38972661 PMC11436954

[21] Svendsen MJ Nicholl BI Mair FS Wood K Rasmussen CDN Stochkendahl MJ. One size does not fit all: participants’ experiences of the selfBACK app to support self-management of low back pain—a qualitative interview study. Chiropractic Manual Ther. (2022) 30:41. doi: 10.1186/s12998-022-00452-2, 36192724 PMC9531397

[22] Lewis J Ridehalgh C Moore A Hall K. This is the day your life must surely change. Physiotherapy. (2021) 112:158–62. doi: 10.1016/j.physio.2021.05.007, 34111808

[23] Roose E Nijs J Moseley GL. Striving for better outcomes of treating chronic pain: integrating behavioural change strategies before, during, and after modern pain science education. Braz J Phys Ther. (2023) 27:100578. doi: 10.1016/j.bjpt.2023.100578, 38096753 PMC10727936

[24] Hooten WM Backonja M Williams KA Sturgeon JA Gross JB Borodianski S . Integrated pain care models and the importance of aligning stakeholder values. PAIN Rep. (2024) 9:e1160. doi: 10.1097/PR9.0000000000001160, 38646660 PMC11029933

[25] Wang H-Y Grech C Evans D Jayasekara R. Education programs for people living with chronic pain: a scoping review. Front Nurs. (2020) 7:307–19. doi: 10.2478/fon-2020-0040

[26] Piette JD Newman S Krein SL Marinec N Chen J Williams DA . Patient-centered pain care using artificial intelligence and Mobile health tools: a randomized comparative effectiveness trial. JAMA Intern Med. (2022) 182:975–83. doi: 10.1001/jamainternmed.2022.3178, 35939288 PMC9361183

[27] Barreveld AM Rosén Klement ML Cheung S Axelsson U Basem JI Reddy AS . An artificial intelligence-powered, patient-centric digital tool for self-management of chronic pain: a prospective, multicenter clinical trial. Pain Med. (2023) 24:1100–10. doi: 10.1093/pm/pnad049, 37104747

[28] Kuwabara A Su S Krauss J. Utilizing digital health Technologies for Patient Education in lifestyle medicine. Am J Lifestyle Med. (2020) 14:137–42. doi: 10.1177/1559827619892547, 32231478 PMC7092400

[29] Devan H Hale L Hempel D Saipe B Perry MA. What works and does not work in a self-management intervention for people with chronic pain? Qualitative systematic review and Meta-synthesis. Phys Ther. (2018) 98:381–97. doi: 10.1093/ptj/pzy029, 29669089

[30] Martinez-Calderon J Flores-Cortes M Morales-Asencio JM Fernandez-Sanchez M Luque-Suarez A. Which interventions enhance pain self-efficacy in people with chronic musculoskeletal pain? A systematic review with Meta-analysis of randomized controlled trials, including over 12 000 participants. J Orthop Sports Phys Ther. (2020) 50:418–30. doi: 10.2519/jospt.2020.9319, 32736497

[31] Baker MB Liu EC Bully MA Hsieh A Nozari A Tuler M . Overcoming barriers: a comprehensive review of chronic pain management and accessibility challenges in rural America. Healthcare. (2024) 12:1765. doi: 10.3390/healthcare12171765, 39273789 PMC11394986

[32] Robinson C D’Souza R Yazdi C Diejomaoh E Schatman M Emerick T . Reviewing the potential role of artificial intelligence in delivering personalized and interactive pain medicine education for chronic pain patients. J Pain Res. (2024) 17:923–9. doi: 10.2147/JPR.S439452 PMC10924768 38464902

[33] Tricco AC Lillie E Zarin W O’Brien KK Colquhoun H Levac D . PRISMA extension for scoping reviews (PRISMA-ScR): checklist and explanation. Ann Intern Med. (2018) 169:467–73. doi: 10.7326/M18-0850, 30178033

[34] Peters MDJ Godfrey CM Khalil H McInerney P Parker D Soares CB. Guidance for conducting systematic scoping reviews. Int J Evid Based Healthc. (2015) 13:141–6. doi: 10.1097/XEB.0000000000000050, 26134548

[35] Davidhizar R Bechtel G Dowd SB. Patient education: a mandate for health care in the 21st century. J Nucl Med Technol. (1998) 26:235–241; quiz 243. 9884934

[36] World Health Organisation. Therapeutic Patient Education: An Introductory Guide. Geneva: World Health Organisation (2023).

[37] Hauser-Ulrich S Künzli H Meier-Peterhans D Kowatsch T. A smartphone-based health care Chatbot to promote self-Management of Chronic Pain (SELMA): pilot randomized controlled trial. JMIR Mhealth Uhealth. (2020) 8:e15806. doi: 10.2196/15806, 32242820 PMC7165314

[38] Bramer WM Rethlefsen ML Kleijnen J Franco OH. Optimal database combinations for literature searches in systematic reviews: a prospective exploratory study. Syst Rev. (2017) 6:245. doi: 10.1186/s13643-017-0644-y, 29208034 PMC5718002

[39] O’Neill J Tabish H Welch V Petticrew M Pottie K Clarke M . Applying an equity lens to interventions: using PROGRESS ensures consideration of socially stratifying factors to illuminate inequities in health. J Clin Epidemiol. (2014) 67:56–64. doi: 10.1016/j.jclinepi.2013.08.005, 24189091

[40] Ali SM Mountain DC Lee RR Murphy D Chiarotto A Wong DC . The current state of digital manikins to support pain self-reporting: a systematic literature review. PAIN Rep. (2025) 10:e1274. doi: 10.1097/PR9.0000000000001274, 40342625 PMC12058649

[41] Sendak MP D’Arcy J Kashyap S Gao M Nichols M Corey K . A path for translation of machine learning products into healthcare delivery. EMJ Innov. (2020). doi: 10.33590/emjinnov/19-00172, 41902734

[42] Bosma R Bisson EJ Cooper LK Salomons TV Galica J Wilson R. Experience-based design: empowering individuals while they wait for interprofessional chronic pain care. Patient Educ Couns. (2023) 109:107623. doi: 10.1016/j.pec.2023.107623, 36640452

[43] Caiata Zufferey M Schulz PJ. Self-management of chronic low back pain: an exploration of the impact of a patient-centered website. Patient Educ Couns. (2009) 77:27–32. doi: 10.1016/j.pec.2009.01.016, 19321286

[44] Camerini L Camerini A-L Schulz PJ. Do participation and personalization matter? A model-driven evaluation of an internet-based patient education intervention for fibromyalgia patients. Patient Educ Couns. (2013) 92:229–34. doi: 10.1016/j.pec.2013.04.007, 23664234

[45] Camerini L Schulz PJ. Effects of functional interactivity on patients’ knowledge, empowerment, and health outcomes: an experimental model-driven evaluation of a web-based intervention. J Med Internet Res. (2012) 14:e105. doi: 10.2196/jmir.1953, 22810046 PMC3409610

[46] Costa F Janela D Molinos M Moulder RG Lains J Francisco GE . Digital rehabilitation for hand and wrist pain: a single-arm prospective longitudinal cohort study. PAIN Rep. (2022) 7:e1026. doi: 10.1097/PR9.0000000000001026, 36003064 PMC9394689

[47] DasMahapatra P Chiauzzi E Pujol LM Los C Trudeau KJ. Mediators and moderators of chronic pain outcomes in an online self-management program. Clin J Pain. (2015) 31:404–13. doi: 10.1097/AJP.0000000000000125, 24918473 PMC4262714

[48] Dear BF Gandy M Karin E Fogliati R Fogliati VJ Staples LG . The pain course: 12- and 24-month outcomes from a randomized controlled trial of an internet-delivered pain management program provided with different levels of clinician support. J Pain. (2018) 19:1491–503. doi: 10.1016/j.jpain.2018.07.005, 30099209

[49] Eilayyan O Thomas A Hallé M-C Ahmed S Tibbles AC Jacobs C . Promoting the use of self-management in patients with spine pain managed by chiropractors and chiropractic interns: barriers and design of a theory-based knowledge translation intervention. Chiropr Man Ther. (2019) 27:44. doi: 10.1186/s12998-019-0267-6, 31636895 PMC6794734

[50] Feldberg G Ricciardi JBS Zorzi AR Yamaguti-Hayakawa GG Ozelo MC. Promoting pain coping skills in haemophilia: a remote intervention integrating exercise and pain education. Haemophilia. (2024) 30:800–8. doi: 10.1111/hae.14991, 38523258

[51] Geraghty AWA Becque T Roberts LC Hill JC Foster NE Yardley L . Supporting self-management of low back pain with an internet intervention with and without telephone support in primary care (SupportBack 2): a randomised controlled trial of clinical and cost-effectiveness. Lancet Rheumatol. (2024) 6:e424–37. doi: 10.1016/S2665-9913(24)00086-9, 38824934

[52] Hearn JH Finlay KA. Internet-delivered mindfulness for people with depression and chronic pain following spinal cord injury: a randomized, controlled feasibility trial. Spinal Cord. (2018) 56:750–61. doi: 10.1038/s41393-018-0090-2, 29581519

[53] Higgins DM Buta E Williams DA Halat A Bair MJ Heapy AA . Internet-based pain self-Management for Veterans: feasibility and preliminary efficacy of the pain EASE program. Pain Pract. (2020) 20:357–70. doi: 10.1111/papr.12861, 31778281

[54] Knudsen LR Ndosi M Hauge E-M Lomborg K Dreyer L Aaboe S . Effectiveness of a novel digital patient education programme to support self-management of early rheumatoid arthritis: a randomized controlled trial. Rheumatology. (2024) 63:2547–56. doi: 10.1093/rheumatology/keae177, 38498833

[55] O’Brien-Kelly J Moore D O’Leary I O’Connor T Moore Z Patton D . Development and impact of a tailored eHealth resource on fibromyalgia patient’s self-management and self-efficacy: a mixed methods approach. Br J Pain. (2024) 18:292–307. doi: 10.1177/20494637231221647, 38751562 PMC11092935

[56] Özden F Özkeskin M Tümtürk İ Yalın Kılınç C. The effect of exercise and education combination via telerehabilitation in patients with chronic neck pain: a randomized controlled trial. Int J Med Inform. (2023) 180:105281. doi: 10.1016/j.ijmedinf.2023.105281, 37924590

[57] Peters ML Smeets E Feijge M Van Breukelen G Andersson G Buhrman M . Happy despite pain: a randomized controlled trial of an 8-week internet-delivered positive psychology intervention for enhancing well-being in patients with chronic pain. Clin J Pain. (2017) 33:962–75. doi: 10.1097/AJP.0000000000000494, 28379873 PMC5636048

[58] Riva S Camerini A-L Allam A Schulz PJ. Interactive sections of an internet-based intervention increase empowerment of chronic Back pain patients: randomized controlled trial. J Med Internet Res. (2014) 16:e180. doi: 10.2196/jmir.3474, 25119374 PMC4147711

[59] Schulz PJ Rubinell S Hartung U. An internet-based approach to enhance self-management of chronic low back pain in the Italian-speaking population of Switzerland: results from a pilot study. Int J Public Health. (2007) 52:286–94. doi: 10.1007/s00038-007-5127-9, 18030944

[60] Schulz PJ Rubinelli S Mariotti G Keller N. Meeting the ranging of informational needs of chronic low back pain sufferers: conceptual design and rationale of the interactive website ONESELF. Disabil Rehabil. (2003) 25:2118–24. doi: 10.3109/09638280902943231, 19888842

[61] Schulz PJ Rubinelli S Zufferey MC Hartung U. Coping with chronic lower back pain: designing and testing the online tool ONESELF. J Comput-Mediat Commun. (2010) 15:625–45. doi: 10.1111/j.1083-6101.2009.01509.x

[62] Srikesavan C Williamson E Lamb SE. The online version of an evidence-based hand exercise programme for people with rheumatoid arthritis: an effectiveness-implementation study. J Hand Ther. (2025) 38:23–32. doi: 10.1016/j.jht.2024.11.001, 39753498

[63] Wilson M Finlay M Orr M Barbosa-Leiker C Sherazi N Roberts MLA . Engagement in online pain self-management improves pain in adults on medication-assisted behavioral treatment for opioid use disorders. Addict Behav. (2018) 86:130–7. doi: 10.1016/j.addbeh.2018.04.019, 29731244

[64] Bostrøm K Børøsund E Varsi C Eide H Flakk Nordang E Schreurs KM . Digital self-Management in Support of patients living with chronic pain: feasibility pilot study. JMIR Form Res. (2020) 4:e23893. doi: 10.2196/23893, 33094734 PMC7647816

[65] Kang SG Lee EN. Development and evaluation of a self-management application for patients with gout. Jpn J Nurs Sci. (2020) 17:e12285. doi: 10.1111/jjns.12285, 31642172

[66] Kiadaliri A Hörder H Lohmander LS Dahlberg LE. Weekly pain trajectories among people with knee or hip osteoarthritis participating in a digitally delivered first-line exercise and education treatment. Pain Med. (2024) 25:291–9. doi: 10.1093/pm/pnad167, 38127991

[67] Mahmoudian A Lohmander LS Dahlberg LE Kiadaliri A. Digital self-management, analgesic use, and patient-reported outcomes in knee or hip osteoarthritis. Arch Phys Med Rehabil. (2024) 105:1821–8. doi: 10.1016/j.apmr.2024.05.033, 38866226

[68] Mohabbat AB Salonen BR Davis XD Volcheck MM Luedtke CA Natividad LT . In-person or virtual educational preferences in patients with fibromyalgia: a cross-sectional survey study at an Academic Medical Center. Pain Manag Nurs. (2024) 25:389–94. doi: 10.1016/j.pmn.2024.03.007, 38570215

[69] Nes AAG Van Dulmen S Wicksell R Fors EA Eide H. Analyzing change processes resulting from a smartphone maintenance intervention based on acceptance and commitment therapy for women with chronic widespread pain. Int J Behav Med. (2017) 24:215–29. doi: 10.1007/s12529-016-9590-7, 27541314

[70] Oh H Suh C Kim J Boo S. Mhealth-based self-management program for patients with rheumatoid arthritis: a pilot randomized controlled study. Nurs Health Sci. (2024) 26:e13187. doi: 10.1111/nhs.13187, 39498761 PMC11617501

[71] Selter A Tsangouri C Ali SB Freed D Vatchinsky A Kizer J . An mHealth app for self-Management of Chronic Lower Back Pain (Limbr): pilot study. JMIR Mhealth Uhealth. (2018) 6:e179. doi: 10.2196/mhealth.8256, 30224333 PMC6231870

[72] Slater H Stinson JN Jordan JE Chua J Low B Lalloo C . Evaluation of digital technologies tailored to support young people’s self-Management of Musculoskeletal Pain: mixed methods study. J Med Internet Res. (2020) 22:e18315. doi: 10.2196/18315, 32442143 PMC7305555

[73] Strand EB Varsi C Børøsund E Eide H Schreurs KMG Waxenberg LB . Changes in cognition, coping, pain and emotions after 12-months access to the digital self-management program EPIO. Front Psychol. (2025) 16:1540852. doi: 10.3389/fpsyg.2025.1540852, 40070899 PMC11893590

[74] Wong AKC Bayuo J Wong FKY Chow KKS Wong SM Lau ACK. The synergistic effect of nurse proactive phone calls with an mHealth app program on sustaining app usage: 3-arm randomized controlled trial. J Med Internet Res. (2023) 25:e43678. doi: 10.2196/43678, 37126378 PMC10186190

[75] Yilmaz E Polat Ü. Online disease management training for older adults with osteoarthritis: a randomized controlled trial. Musculoskeletal Care. (2024) 22:e1931. doi: 10.1002/msc.1931, 39134868

[76] Deegan O Fullen BM Hearty C Doody CM. Acceptability of the combined online interactive mindfulness and exercise programme (MOVE-online) for adults with chronic pain – a qualitative study. Disabil Rehabil. (2024) 46:4394–406. doi: 10.1080/09638288.2023.2274875, 37886895

[77] Carmody TP Duncan CL Huggins J Solkowitz SN Lee SK Reyes N . Telephone-delivered cognitive–behavioral therapy for pain management among older military veterans: a randomized trial. Psychol Serv. (2013) 10:265–75. doi: 10.1037/a0030944, 23244028

[78] Gray E Erickson M Bindler R Eti DU Wilson M. Experiencing COMFORT: perceptions of virtually-delivered nonpharmacologic therapies in adults prescribed opioids for chronic pain. Pain Manag Nurs. (2023) 24:469–76. doi: 10.1016/j.pmn.2023.04.002, 37179236 PMC10524526

[79] Grazzi L Montisano DA D’Amico D Altamura C Raggi A Rizzoli P . Multimodal digital health treatments for chronic migraine associated with medication overuse headache: a literature appraisal and results of a single-arm open trial (the BE-HOME program). Neurol Sci. (2024) 45:4923–30. doi: 10.1007/s10072-024-07568-8, 38771524

[80] Kennedy CA Warmington K Flewelling C Shupak R Papachristos A Jones C . A prospective comparison of telemedicine versus in-person delivery of an interprofessional education program for adults with inflammatory arthritis. J Telemed Telecare. (2017) 23:197–206. doi: 10.1177/1357633X16635342, 26957111

[81] Post AA Rio EK Sluka KA Moseley GL Bayman EO Hall MM . Efficacy of telehealth for movement-evoked pain in people with chronic Achilles tendinopathy: a noninferiority analysis. Phys Ther. (2023) 103:pzac171. doi: 10.1093/ptj/pzac171, 37172125 PMC10071585

[82] Song Y Reifsnider E Zhao S Xie X Chen H. A randomized controlled trial of the effects of a telehealth educational intervention on medication adherence and disease activity in rheumatoid arthritis patients. J Adv Nurs. (2020) 76:1172–81. doi: 10.1111/jan.14319, 32026506

[83] Warmington K Flewelling C Kennedy C Shupak R Papachristos A Jones C . Telemedicine delivery of patient education in remote Ontario communities: feasibility of an advanced clinician practitioner in arthritis care (ACPAC)-led inflammatory arthritis education program. Open Access Rheumatol. (2017) 9:11–9. doi: 10.2147/OARRR.S122015, 28280400 PMC5338940

[84] Zhao S Chen H. Effectiveness of health education by telephone follow-up on self-efficacy among discharged patients with rheumatoid arthritis: a randomised control trial. J Clin Nurs. (2019) 28:3840–7. doi: 10.1111/jocn.15002, 31325348

[85] Perry J VanDenKerkhof EG Wilson R Tripp DA. Guided internet-based psycho-educational intervention using cognitive behavioral therapy and self-management for individuals with chronic pain: a feasibility study. Pain Manag Nurs. (2017) 18:179–89. doi: 10.1016/j.pmn.2016.12.003, 28433488

[86] Brunner WM Pullyblank K Scribani MB Krupa N Wyckoff L. Remote delivery of self-management education workshops for adults with chronic pain, 2020–2021. Chronic Illn. (2024) 20:360–8. doi: 10.1177/17423953231181408, 37291805

[87] Elander J Robinson G Morris J. Randomized trial of a DVD intervention to improve readiness to self-manage joint pain. Pain. (2011) 152:2333–41. doi: 10.1016/j.pain.2011.06.026, 21820244

[88] Elander J Stalker C. Effects of a pain self-management intervention combining written and video elements on health-related quality of life among people with different levels of education. J Pain Res. (2015) 8:581–90. doi: 10.2147/JPR.S85741, 26316809 PMC4548759

[89] Miller JS Litva A Gabbay M. Motivating patients with shoulder and back pain to self-care: can a videotape of exercise support physiotherapy? Physiotherapy. (2009) 95:29–35. doi: 10.1016/j.physio.2007.07.009, 19627683

[90] Nisar M Khan A Kolbe-Alexander TL. ‘Cost, culture and circumstances’: barriers and enablers of health behaviours in south Asian immigrants of Australia. Health Soc Care Community. (2022) 30:e3138–49. doi: 10.1111/hsc.13759, 35181973 PMC9543603

[91] Shetty S Scuffell J Aitken D Ashworth M. Chronic pain: prevalence, demographic inequalities, and healthcare utilisation. A primary care database analysis. BJGP Open. (2025) 9:147. doi: 10.3399/BJGPO.2024.0147, 40015745 PMC12728837

[92] Vargas A Tobey-Moore L Curran G Elkhateb R Sexton K Bailey B . Exploring racial disparities in chronic pain management. J Pain Res. (2025) 18:2901–8. doi: 10.2147/JPR.S493664, 40520808 PMC12164869

[93] Wong JJ Côté P Tricco AC Watson T Rosella LC. Characterizing high-cost healthcare users among adults with back pain in Ontario, Canada: a population-based cohort study. Pain. (2024) 165:1944–54. doi: 10.1097/j.pain.0000000000003200, 38442409 PMC11331817

[94] Main A McCartney H Ibrar M Muirhead F Mavroeidi A Rai HK . Patients’ experiences of digital health interventions for the self-Management of Chronic Pain: systematic review and thematic synthesis. J Med Internet Res. (2025) 27:e69100. doi: 10.2196/69100, 40101209 PMC11962327

[95] Shetty A Delanerolle G Cavalini H Deng C Yang X Boyd A . A systematic review and network meta-analysis of pharmaceutical interventions used to manage chronic pain. Sci Rep. (2024) 14:1621. doi: 10.1038/s41598-023-49761-3, 38238384 PMC10796361

[96] Best A Saul JE. Systems thinking: a different window on the world of implementation and global exchange of behavioral medicine evidence. Transl Behav Med. (2011) 1:361–3. doi: 10.1007/s13142-011-0050-2, 24073056 PMC3717640

[97] Oldenburg B Absetz P. Lost in translation: overcoming the barriers to global implementation and exchange of behavioral medicine evidence. Transl Behav Med. (2011) 1:252–5. doi: 10.1007/s13142-011-0051-1, 21765879 PMC3120972

[98] Knight H Stetson B Krishnasamy S Mokshagundam SP. Diet self-management and readiness to change in underserved adults with type 2 diabetes. Prim Care Diabetes. (2015) 9:219–25. doi: 10.1016/j.pcd.2014.09.007, 25457433

[99] Barlow J Wright C Sheasby J Turner A Hainsworth J. Self-management approaches for people with chronic conditions: a review. Patient Educ Couns. (2002) 48:177–87. doi: 10.1016/S0738-3991(02)00032-0, 12401421

[100] Ali SM Lee RR McBeth J James B McAlister S Chiarotto A . Exploring the cross-cultural acceptability of digital tools for pain self-reporting: qualitative study. JMIR Hum Factors. (2023) 10:e42177. doi: 10.2196/42177, 36753324 PMC9947768

[101] Ali SM Masood Y Staniland K Dixon WG Van der Veer SN Sanders C. Perspectives on remote monitoring technology of south Asians living with a long-term condition in the United Kingdom: a qualitative study (preprint) (2025) 1:82333. doi: 10.2196/preprints.82333, PMC13211867 42190266

[102] Toonders SAJ van der Meer HA Van Bruxvoort T Veenhof C Speksnijder CM. Effectiveness of remote physiotherapeutic e-health interventions on pain in patients with musculoskeletal disorders: a systematic review. Disabil Rehabil. (2023) 45:3620–38. doi: 10.1080/09638288.2022.2135775, 36369923

[103] Valentijn PP Tymchenko L Jacobson T Kromann J Biermann CW AlMoslemany MA . Digital health interventions for musculoskeletal pain conditions: systematic review and Meta-analysis of randomized controlled trials. J Med Internet Res. (2022) 24:e37869. doi: 10.2196/37869, 36066943 PMC9490534

[104] Lopez-Alcalde J Susan Wieland L Barth J Grainger R Baxter N Heron N . Methodological challenges in systematic reviews of mHealth interventions: survey and consensus-based recommendations. Int J Med Inform. (2024) 184:105345. doi: 10.1016/j.ijmedinf.2024.105345, 38309237 PMC11192046

