# Supplementary material for: Scoping review of equity considerations in digitally enabled patient education interventions for self-managing chronic musculoskeletal pain
Source: Front Public Health. 2026 Sep 30;14:1954179. doi: 10.3389/fpubh.2026.1954179 (PMC13661994; doi:10.3389/fpubh.2026.1954179)
Supplement: Supplementary file 1 [file Data_Sheet_1.PDF]

ANNEXURE A: Search Strategy for Medline

**Search conducted on: 16-01-2025**		
**S.NO**	**Ovid MEDLINE(R) ALL <1946 to January 15, 2025>**	**# of hits**
1	Patient Participation/ or health literacy/ or consumer health information/ or consumer health informatics/ or patient education/ or patient education.mp. or exp patient education/ or Patient Involvement/ or Patient Empowerment/ or Patient Activation/ or Patient Engagement/ or health literacy.mp. or patient guideline*.mp. or teaching material*/ or instructional material*/ or educational material*/ or consumer health information.mp. or consumer health informatics.mp. or consumer health material*/ or Self?care/ or self?care.mp. or self?care skills/ or self?manage*.mp. or self-manage/ or health education/ or health behaviour/ or self manag*/ or self help technique*.mp. or empowerment/ or self efficacy.mp. or self efficacy/	279917
2	(chronic adj2 (pain or primary pain or secondary pain or migraine or headache* or ache)).mp.	93061
3	(Persistent pain* or cancer pain or fibromyalgia or back pain* or sciatica or arthritis or rheumatoid arthritis or osteoarthritis or spondylarthritis or endometriosis or neuropathic pain* or irritable bowel syndrome or complex regional pain syndrome or orofacial pain or chronic primary visceral pain or joint pain* or musculoskeletal pain*).mp.	578644
4	2 or 3	637432
5	1 and 4	9357
6	(health literacy or patient education).mp. or exp patient education/ or Patient Empowerment.mp. or Patient Engagement.mp. or patient guideline*.mp. or Self?care/ or self?care.mp. or self?care skills/ or self?manage*.mp. or self-manage/ or health education/ or self manag*/ or self help technique*.mp. [mp=title, book title, abstract, original title, name of substance word, subject heading word, floating sub-heading word, keyword heading word, organism supplementary concept word, protocol supplementary concept word, rare disease supplementary concept word, unique identifier, synonyms, population supplementary concept word, anatomy supplementary concept word]	206577
7	4 and 6	6466
8	(Persistent pain or fibromyalgia or back pain or sciatica or arthritis or rheumatoid arthritis or osteoarthritis or spondylarthritis or endometriosis or neuropathic pain or complex regional pain syndrome or joint pain or musculoskeletal pain).mp. [mp=title, book title, abstract, original title, name of substance word, subject heading word, floating sub-heading word, keyword heading word, organism supplementary concept word, protocol supplementary concept word, rare disease supplementary concept word, unique identifier, synonyms, population supplementary concept word, anatomy supplementary concept word]	547429
9	2 or 8	609380
10	6 and 9	5965

11	((chronic adj2 (pain or primary pain or secondary pain or migraine or headache* or ache)) or Persistent pain or fibromyalgia or back pain or arthritis or neuropathic pain or complex regional pain syndrome or joint pain or musculoskeletal pain).mp. [mp=title, book title, abstract, original title, name of substance word, subject heading word, floating sub-heading word, keyword heading word, organism supplementary concept word, protocol supplementary concept word, rare disease supplementary concept word, unique identifier, synonyms, population supplementary concept word, anatomy supplementary concept word]	475763
12	6 and 11	5043

ANNEXURE B: List of included studies

1. Areias AC, Janela D, Moulder RG, Molinos M, Bento V, Moreira C, Yanamadala V, Correia FD, Costa F. Applying AI to safely and effectively scale care to address chronic MSK conditions. Journal of Clinical Medicine. 2024 Jul 26;13(15):4366.
2. Allen KD, Oddone EZ, Coffman CJ, Datta SK, Juntilla KA, Lindquist JH, Walker TA, Weinberger M, Bosworth HB. Telephone-based self-management of osteoarthritis: a randomized trial. Annals of internal medicine. 2010 Nov 2;153(9):570-9.
3. Appel PR, Bleiberg J, Noiseux J. Self-regulation training for chronic pain: can it be done effectively by telemedicine?. Telemedicine Journal and e-Health. 2002 Dec 1;8(4):361-8.
4. Brunner WM, Pullyblank K, Scribani MB, Krupa N, Wyckoff L. Remote delivery of self-management education workshops for adults with chronic pain, 2020–2021. Chronic Illness. 2024 Jun;20(2):360-8.
5. Barreveld AM, Rosén Klement ML, Cheung S, Axelsson U, Basem JI, Reddy AS, Borrebaeck CA, Mehta N. An artificial intelligence-powered, patient-centric digital tool for self-management of chronic pain: a prospective, multicenter clinical trial. Pain Medicine. 2023 Sep 1;24(9):1100-10.
6. Bosma R, Bisson EJ, Cooper LK, Salomons TV, Galica J, Wilson R. Experience-based design: Empowering individuals while they wait for interprofessional chronic pain care. Patient Education and Counseling. 2023 Apr 1;109:107623.
7. Brown L, DiCenso-Fleming T, Ensign T, Boyd AJ, Monaghan G, Binder DS. Chronic pain education delivered with a virtual reality headset in outpatient physical therapy clinics: a multi-site exploratory trial. American journal of translational research. 2023 May 15;15(5):3500
8. Bennell KL, Nelligan R, Dobson F, Rini C, Keefe F, Kasza J, French S, Bryant C, Dalwood A, Abbott JH, Hinman RS. Effectiveness of an internet-delivered exercise and pain-coping skills training intervention for persons with chronic knee pain: a randomized trial. Annals of internal medicine. 2017 Apr 4;166(7):453-62.

9. Bromberg J, Wood ME, Black RA, Surette DA, Zacharoff KL, Chiauzzi EJ. A randomized trial of a web-based intervention to improve migraine self-management and coping. Headache: The Journal of Head and Face Pain. 2012 Feb;52(2):244-61.
10. Berman RL, Iris MA, Bode R, Drengenberg C. The effectiveness of an online mind-body intervention for older adults with chronic pain. The journal of Pain. 2009 Jan 1;10(1):68-79
11. Bostrøm K, Børøsund E, Varsi C, Eide H, Nordang EF, Schreurs KM, Waxenberg LB, Weiss KE, Morrison EJ, Småstuen MC, Stubhaug A. Digital self-management in support of patients living with chronic pain: feasibility pilot study. JMIR Formative Research. 2020 Oct 23;4(10):e23893.
12. Calner T, Nordin C, Eriksson MK, Nyberg L, Gard G, Michaelson P. Effects of a self-guided, web-based activity programme for patients with persistent musculoskeletal pain in primary healthcare: A randomized controlled trial. European journal of pain. 2017 Jul;21(6):1110-20.
13. Camerini L, Camerini AL, Schulz PJ. Do participation and personalization matter? A model-driven evaluation of an Internet-based patient education intervention for fibromyalgia patients. Patient education and counseling. 2013 Aug 1;92(2):229-34.
14. Carmody TP, Duncan CL, Huggins J, Solkowitz SN, Lee SK, Reyes N, Mozgai S, Simon JA. Telephone-delivered cognitive–behavioral therapy for pain management among older military veterans: A randomized trial. Psychological Services. 2013 Aug;10(3):265.
15. Camerini L, Schulz PJ. Effects of functional interactivity on patients' knowledge, empowerment, and health outcomes: An experimental model-driven evaluation of a web-based intervention. Journal of medical Internet research. 2012 Jul 18;14(4):e1953.
16. Carpenter KM, Stoner SA, Mundt JM, Stoelb B. An online self-help CBT intervention for chronic lower back pain. The Clinical journal of pain. 2012 Jan 1;28(1):14-22.
17. Chiauzzi E, Pujol LA, Wood M, Bond K, Black R, Yiu E, Zacharoff K. painACTION-back pain: a self-management website for people with chronic back pain. Pain Medicine. 2010 Jul 1;11(7):1044-58.
18. Costa F, Janela D, Molinos M, Moulder RG, Lains J, Francisco GE, Bento V, Yanamadala V, Cohen SP, Correia FD. Digital rehabilitation for hand and wrist pain: a single-arm prospective longitudinal cohort study. Pain Reports. 2022 Sep 1;7(5):e1026.
19. de Bruijn-Kofman AT, Van de Wiel H, Groenman NH, Sorbi MJ, Klip E. Effects of a mass media behavioral treatment for chronic headache: A pilot study. Headache: The Journal of Head and Face Pain. 1997 Jul;37(7):415-20.
20. Duggan GB, Keogh E, Mountain GA, McCullagh P, Leake J, Eccleston C. Qualitative evaluation of the SMART2 self-management system for people in chronic pain. Disability and Rehabilitation: Assistive Technology. 2015 Jan 2;10(1):53-60.

21. Dowd H, Hogan MJ, McGuire BE, Davis MC, Sarma KM, Fish RA, Zautra AJ. Comparison of an online mindfulness-based cognitive therapy intervention with online pain management psychoeducation: a randomized controlled study. The Clinical journal of pain. 2015 Jun 1;31(6):517-27.
22. DasMahapatra P, Chiauzzi E, Pujol LM, Los C, Trudeau KJ. Mediators and moderators of chronic pain outcomes in an online self-management program. The Clinical journal of pain. 2015 May 1;31(5):404-13.
23. Deegan O, Fullen BM, Hearty C, Doody CM. Acceptability of the combined online interactive mindfulness and exercise programme (MOVE-Online) for adults with chronic pain–A qualitative study. Disability and Rehabilitation. 2024 Sep 10;46(19):4394-406.
24. de Vries FS, van Dongen RT, Bertens D. Pain education and pain management skills in virtual reality in the treatment of chronic low back pain: A multiple baseline single-case experimental design. Behaviour Research and Therapy. 2023 Mar 1;162:104257.
25. Dear BF, Gandy M, Karin E, Fogliati R, Fogliati VJ, Staples LG, Wootton BM, Sharpe L, Titov N. The pain course: 12-and 24-month outcomes from a randomized controlled trial of an internet-delivered pain management program provided with different levels of clinician support. The Journal of Pain. 2018 Dec 1;19(12):1491-503.
26. Elander J, Robinson G, Morris J. Randomized trial of a DVD intervention to improve readiness to self-manage joint pain. PAIN®. 2011 Oct 1;152(10):2333-41
27. Egerton T, McLachlan L, Graham B, Bolton J, Setchell J, Short CE, Bryant C, Bennell KL. How do people with knee pain from osteoarthritis respond to a brief video delivering empowering education about the condition and its management?. Patient Education and Counseling. 2021 Aug 1;104(8):2018-27.
28. Eilayyan O, Thomas A, Hallé MC, Ahmed S, Tibbles AC, Jacobs C, Mior S, Davis C, Evans R, Schneider MJ, Owens H. Promoting the use of self-management in patients with spine pain managed by chiropractors and chiropractic interns: barriers and design of a theory-based knowledge translation intervention. Chiropractic & Manual Therapies. 2019 Oct 16;27(1):44.
29. Fritsch CG, Abdel-Shaheed C, Mohamed R, Ferreira PH, McLachlan AJ, Ferreira ML. A qualitative assessment of a text message intervention for people with low back pain. Musculoskeletal Science and Practice. 2023 Apr 1;64:102739.
30. Feldberg G, Ricciardi JB, Zorzi AR, Yamaguti-Hayakawa GG, Ozelo MC. Promoting pain coping skills in haemophilia: a remote intervention integrating exercise and pain education. Haemophilia. 2024 May;30(3):800-8.

31. Grazzi L, Montisano DA, D’Amico D, Altamura C, Raggi A, Rizzoli P, Marcassoli A. Multimodal digital health treatments for Chronic Migraine associated with Medication Overuse Headache: a literature appraisal and results of a single-arm open trial (the BE-HOME program). Neurological Sciences. 2024 Oct;45(10):4923-30.
32. Geraghty AW, Becque T, Roberts LC, Hill JC, Foster NE, Yardley L, Stuart B, Turner DA, Hay E, Griffiths G, Webley F. Supporting self-management of low back pain with an internet intervention with and without telephone support in primary care (SupportBack 2): a randomised controlled trial of clinical and cost-effectiveness. The Lancet Rheumatology. 2024 Jul 1;6(7):e424-37.
33. Garvin L, Driscoll MA, Steffensmeier KS, Johnson NL, Adamowicz JL, Obrecht AA, Hart KJ, Rothmiller SJ, Sibenaller Z, Stout L, Richards C. Targeting chronic pain care to rural women veterans: A feasibility pilot. Psychological services. 2024 Oct 7.
34. Gray E, Erickson M, Bindler R, Eti DU, Wilson M. Experiencing COMFORT: Perceptions of virtually-delivered nonpharmacologic therapies in adults prescribed opioids for chronic pain. Pain Management Nursing. 2023 Aug 1;24(4):469-76.
35. Higgins DM, Buta E, Williams DA, Halat A, Bair MJ, Heapy AA, Krein SL, Rajeevan H, Rosen MI, Kerns RD. Internet-Based Pain Self-Management for Veterans: Feasibility and Preliminary Efficacy of the Pain EASE Program. Pain Practice. 2020 Apr;20(4):357-70.
36. Hearn JH, Finlay KA. Internet-delivered mindfulness for people with depression and chronic pain following spinal cord injury: a randomized, controlled feasibility trial. Spinal cord. 2018 Aug;56(8):750-61.
37. Heapy AA, Higgins DM, Goulet JL, LaChappelle KM, Driscoll MA, Czlapinski RA, Buta E, Piette JD, Krein SL, Kerns RD. Interactive voice response–based self-management for chronic back pain: the COPES noninferiority randomized trial. JAMA Internal Medicine. 2017 Jun 1;177(6):765-73.
38. Hutting N, Detaille SI, Heerkens YF, Engels JA, Staal JB, Nijhuis-van der Sanden MW. Experiences of participants in a self-management program for employees with complaints of the arm, neck or shoulder (CANS): a mixed methods study. Journal of occupational rehabilitation. 2017 Mar;27(1):35-48.
39. Hutting N, Staal JB, Engels JA, Heerkens YF, Detaille SI, Nijhuis-van der Sanden MW. Effect evaluation of a self-management programme for employees with complaints of the arm, neck or shoulder: a randomised controlled trial. Occupational and Environmental Medicine. 2015 Dec 1;72(12):852-61.

40. Jay K, Schraefel MC, Brandt M, Andersen LL. Effect of Video-Based versus Personalized Instruction on Errors during Elastic Tubing Exercises for Musculoskeletal Pain: A Randomized Controlled Trial. BioMed research international. 2014;2014(1):790937.
41. Kiadaliri A, Hörder H, Lohmander LS, Dahlberg LE. Weekly pain trajectories among people with knee or hip osteoarthritis participating in a digitally delivered first-line exercise and education treatment. Pain Medicine. 2024 Apr 1;25(4):291-9.
42. Kratz AL, Alschuler KN, Williams DA, Ehde DM. Development and pilot testing of a web-based symptom management program for multiple sclerosis: My MS toolkit. Rehabilitation Psychology. 2021 May;66(2):224.
43. Kang SG, Lee EN. Development and evaluation of a self-management application for patients with gout. Japan Journal of Nursing Science. 2020 Apr;17(2):e12285.
44. Kennedy CA, Warmington K, Flewelling C, Shupak R, Papachristos A, Jones C, Linton D, Beaton DE, Lineker S, Hogg-Johnson S. A prospective comparison of telemedicine versus in-person delivery of an interprofessional education program for adults with inflammatory arthritis. Journal of telemedicine and telecare. 2017 Feb;23(2):197-206.
45. Kumar K, John H, Gordhan C, Situnayake D, Raza K, Bacon PA. Breaking communication barriers for RA patients of South Asian origin: the use of a bilingual educational audio CD and linguistically appropriate peer support and education. Musculoskeletal Care. 2011 Mar;9(1):11-8.
46. Knudsen LR, Lomborg K, Hauge EM, Zangi HA, de Thurah A. The WebRA study: opportunities and challenges in digital patient education from the perspective of patients with rheumatoid arthritis: a qualitative study. Patient Education and Counseling. 2023 Nov 1;116:107969.
47. Knudsen LR, Ndosi M, Hauge EM, Lomborg K, Dreyer L, Aaboe S, Kjær MB, Sørensen L, Volsmann L, Christensen HM, De Thurah A. Effectiveness of a novel digital patient education programme to support self-management of early rheumatoid arthritis: a randomized controlled trial. Rheumatology. 2024 Sep;63(9):2547-56.
48. Lorig KR, Laurent DD, Deyo RA, Marnell ME, Minor MA, Ritter PL. Can a Back Pain E-mail Discussion Group improve health status and lower health care costs?: A randomized study. Archives of internal medicine. 2002 Apr 8;162(7):792-6.
49. Lopez-Olivo MA, Lin H, Rizvi T, Barbo Barthel A, Ingleshwar A, des Bordes JK, Jibaja-Weiss M, Volk RJ, Suarez-Almazor ME. Randomized controlled trial of patient education tools for patients with rheumatoid arthritis. Arthritis care & research. 2021 Oct;73(10):1470-8.
50. Lopez-Olivo MA, Ingleshwar A, Volk RJ, Jibaja-Weiss M, Barbo A, Saag K, Leong A, Suarez-Almazor ME. Development and pilot testing of multimedia patient education tools for patients

with knee osteoarthritis, osteoporosis, and rheumatoid arthritis. Arthritis care & research. 2018 Feb;70(2):213-20.

51. Miller JS, Litva A, Gabbay M. Motivating patients with shoulder and back pain to self-care: can a videotape of exercise support physiotherapy?. Physiotherapy. 2009 Mar 1;95(1):29-35.
52. Modarresi S, Pearson N, Madden K, Fahnestock M, Bowdish D, Carlesso LC. Feasibility of pain informed movement program for people with knee osteoarthritis. Osteoarthritis and Cartilage Open. 2023 Dec 1;5(4):100401.
53. Marcuzzi A, Nordstoga AL, Bach K, Aasdahl L, Nilsen TI, Bardal EM, Boldermo NØ, Bertheussen GF, Marchand GH, Gismervik S, Mork PJ. Effect of an artificial intelligence–based self-management app on musculoskeletal health in patients with neck and/or low back pain referred to specialist care: a randomized clinical trial. JAMA network open. 2023 Jun 1;6(6):e2320400-.
54. Marier-Deschenes P, Pinard AM, Jalbert L, LeBlanc A. Assessing the Feasibility and Preliminary Effects of a Web-Based Self-Management Program for Chronic Noncancer Pain: Mixed Methods Study. JMIR human factors. 2024 May 3;11:e50747.
55. Mohabbat AB, Salonen BR, Davis XD, Volcheck MM, Luedtke CA, Natividad LT, Guzman TD, Johnson SM, Ledvina AJ, Merza CT, Wight EC. In-Person or Virtual Educational Preferences in Patients With Fibromyalgia: A Cross-Sectional Survey Study at an Academic Medical Center. Pain Management Nursing. 2024 Aug 1;25(4):389-94.
56. Mahmoudian A, Lohmander LS, Dahlberg LE, Kiadaliri A. Digital self-management, analgesic use, and patient-reported outcomes in knee or hip osteoarthritis. Archives of physical medicine and rehabilitation. 2024 Oct 1;105(10):1821-8.
57. Morcillo-Muñoz Y, Sánchez-Guarnido AJ, Calzón-Fernández S, Baena-Parejo I. Multimodal chronic pain therapy for adults via smartphone: randomized controlled clinical trial. Journal of Medical Internet Research. 2022 May 11;24(5):e36114.
58. McDonald DD, Walsh S, Vergara C, Gifford T, Weiner DK. The effect of a Spanish virtual pain coach for older adults: A pilot study. Pain Medicine. 2012 Nov 1;13(11):1397-406.
59. Nordstoga AL, Aasdahl L, Sandal LF, Dalager T, Kongsvold A, Mork PJ, Nilsen TI. The role of pain duration and pain intensity on the effectiveness of app-delivered self-management for low back pain (selfBACK): secondary analysis of a randomized controlled trial. JMIR mHealth and uHealth. 2023 Aug 31;11:e40422.
60. Nes AA, van Dulmen S, Wicksell R, Fors EA, Eide H. Analyzing change processes resulting from a smartphone maintenance intervention based on acceptance and commitment therapy for

women with chronic widespread pain. International journal of behavioral medicine. 2017 Apr;24(2):215-29.

61. Nevedal DC, Wang C, Oberleitner L, Schwartz S, Williams AM. Effects of an individually tailored web-based chronic pain management program on pain severity, psychological health, and functioning. Journal of Medical Internet Research. 2013 Sep 25;15(9):e2296.
62. Oh H, Suh CH, Kim JW, Boo S. mHealth-Based Self-Management Program for Patients With Rheumatoid Arthritis: A Pilot Randomized Controlled Study. Nursing & Health Sciences. 2024 Dec;26(4):e13187.
63. O'Brien-Kelly J, Moore D, O'Leary I, O'Connor T, Moore Z, Patton D, Nugent L. Development and impact of a tailored eHealth resource on fibromyalgia patient's self-management and self-efficacy: A mixed methods approach. British Journal of Pain. 2024 Jun;18(3):292-307.
64. Özden F, Özkeskin M, Tümtürk İ, Kılınç CY. The effect of exercise and education combination via telerehabilitation in patients with chronic neck pain: A randomized controlled trial. International Journal of Medical Informatics. 2023 Dec 1;180:105281.
65. Post AA, Rio EK, Sluka KA, Moseley GL, Bayman EO, Hall MM, de Cesar Netto C, Wilken JM, Danielson J, Chimenti RL. Efficacy of telehealth for movement-evoked pain in people with chronic Achilles tendinopathy: a noninferiority analysis. Physical therapy. 2023 Mar 1;103(3):pzac171
66. Pak SS, Janela D, Freitas N, Costa F, Moulder R, Molinos M, Areias AC, Bento V, Cohen SP, Yanamadala V, Souza RB. Comparing digital to conventional physical therapy for chronic shoulder pain: randomized controlled trial. Journal of medical Internet research. 2023 Aug 18;25:e49236.
67. Perry J, VanDenKerkhof EG, Wilson R, Tripp DA. Development of a guided internet-based psycho-education intervention using cognitive behavioral therapy and self-management for individuals with chronic pain. Pain Management Nursing. 2017 Apr 1;18(2):90-101.
68. Perry J, VanDenKerkhof EG, Wilson R, Tripp DA. Guided internet-based psycho-educational intervention using cognitive behavioral therapy and self-management for individuals with chronic pain: a feasibility study. Pain Management Nursing. 2017 Jun 1;18(3):179-89.
69. Poolman EY, Vorstermans L, Donker MH, Bijker L, Coppieters MW, Cuijpers P, Scholten-Peeters GG, de Wit LM. How people with persistent pain experience in-person physiotherapy blended with biopsychosocial digital health-a qualitative study on participants' experiences with Back2Action. Internet Interventions. 2024 Jun 1;36:100731.

70. Pariser D, O'Hanlon A, Espinoza L. Effects of telephone intervention on arthritis self-efficacy, depression, pain, and fatigue in older adults with arthritis. Journal of Geriatric Physical Therapy. 2005 Dec 1;28(3):67-73
71. Peters ML, Smeets E, Feijge M, Van Breukelen G, Andersson G, Buhrman M, Linton SJ. Happy despite pain: a randomized controlled trial of an 8-week internet-delivered positive psychology intervention for enhancing well-being in patients with chronic pain. The Clinical journal of pain. 2017 Nov 1;33(11):962-75.
72. Riva S, Camerini AL, Allam A, Schulz PJ. Interactive sections of an Internet-based intervention increase empowerment of chronic back pain patients: randomized controlled trial. Journal of medical Internet research. 2014 Aug 13;16(8):e3474.
73. Ruehlman LS, Karoly P, Enders C. A randomized controlled evaluation of an online chronic pain self management program. Pain. 2012 Feb 1;153(2):319-30.
74. Srikesavan C, Williamson E, Lamb SE. The online version of an evidence-based hand exercise programme for people with rheumatoid arthritis: An effectiveness-implementation study. Journal of Hand Therapy. 2025 Jan 1;38(1):23-32.
75. Son SJ, Sedgewick J, Lonza G, George J, Li J, Andrawis J. A Video-Based Intervention for Increasing Health Literacy in Total Joint Arthroplasty Patients at a Safety-Net Hospital: A Prospective Single-Blind Cohort Study. Arthroplasty Today. 2024 Dec 1;30:101575.
76. Song Y, Reifsnider E, Zhao S, Xie X, Chen H. A randomized controlled trial of the Effects of a telehealth educational intervention on medication adherence and disease activity in rheumatoid arthritis patients. Journal of advanced nursing. 2020 May;76(5):1172-81.
77. Smith J, Faux SG, Gardner T, Hobbs MJ, James MA, Joubert AE, Kladnitski N, Newby JM, Schultz R, Shiner CT, Andrews G. Reboot online: a randomized controlled trial comparing an online multidisciplinary pain management program with usual care for chronic pain. Pain Medicine. 2019 Dec 1;20(12):2385-96.
78. Selter A, Tsangouri C, Ali SB, Freed D, Vatchinsky A, Kizer J, Sahuguet A, Vojta D, Vad V, Pollak JP, Estrin D. An mHealth app for self-management of chronic lower back pain (Limbr): pilot study. JMIR mHealth and uHealth. 2018 Sep 17;6(9):e8256.
79. Sullivan M, Langford DJ, Davies PS, Tran C, Vilardaga R, Cheung G, Yoo D, McReynolds J, Lober WB, Tauben D, Vowles KE. A controlled pilot trial of PainTracker Self-Manager, a web-based platform combined with patient coaching, to support patients' self-management of chronic pain. The journal of pain. 2018 Sep 1;19(9):996-1005.

80. Stalker C, Elander J. Effects of a pain self-management intervention combining written and video elements on health-related quality of life among people with different levels of education. Journal of pain research. 2015 Aug 20:581-90.
81. Schulz PJ, Rubinelli S, Zufferey MC, Hartung U. Coping with chronic lower back pain: designing and testing the online tool ONESELF. Journal of Computer-Mediated Communication. 2010 Jul 1;15(4):625-45.
82. Schulz PJ, Rubinelli S, Mariotti G, Keller N. Meeting the ranging of informational needs of chronic low back pain sufferers: conceptual design and rationale of the interactive website ONESELF. Disability and rehabilitation. 2009 Jan 1;31(25):2118-24.
83. Schulz PJ, Rubinell S, Hartung U. An internet-based approach to enhance self-management of chronic low back pain in the Italian-speaking population of Switzerland: results from a pilot study. International journal of public health. 2007 Oct;52(5):286-94.
84. Slater H, Stinson JN, Jordan JE, Chua J, Low B, Lalloo C, Pham Q, Cafazzo JA, Briggs AM. Evaluation of digital technologies tailored to support young people's self-management of musculoskeletal pain: mixed methods study. Journal of Medical Internet Research. 2020 Jun 5;22(6):e18315.
85. Strand EB, Varsi C, Børøsund E, Eide H, Schreurs KM, Waxenberg LB, Weiss KE, Morrison EJ, Støle HS, Kristjansdottir ÓB, Stubhaug A. Changes in cognition, coping, pain and emotions after 12-months access to the digital self-management program EPIO. Frontiers in Psychology. 2025 Feb 25;16:1540852.
86. Trudeau KJ, Pujol LA, DasMahapatra P, Wall R, Black RA, Zacharoff K. A randomized controlled trial of an online self-management program for adults with arthritis pain. Journal of behavioral medicine. 2015 Jun;38(3):483-96.
87. Trompetter HR, Bohlmeijer ET, Veehof MM, Schreurs KM. Internet-based guided self-help intervention for chronic pain based on Acceptance and Commitment Therapy: a randomized controlled trial. Journal of behavioral medicine. 2015 Feb;38(1):66-80.
88. Vogler CN, Sattovia S, Salazar LY, Leung TI, Botchway A. Assessing outcomes of educational videos in group visits for patients with chronic pain at an academic primary care clinic. Postgraduate medicine. 2017 Jul 4;129(5):524-30.
89. Window P, McGrath M, Harvie DS, Smits E, Johnston V, Murdoch M, Russell T. Pain Education and Virtual Reality Improves Pain, Pain-related Fear of Movement, and Trunk Kinematics in Individuals With Persistent Low Back Pain. The Clinical Journal of Pain. 2024 Aug 1;40(8):478-89.

90. Wong AK, Bayuo J, Wong FK, Chow KK, Wong SM, Lau AC. The synergistic effect of nurse proactive phone calls with an mHealth app program on sustaining app usage: 3-arm randomized controlled trial. Journal of medical Internet research. 2023 May 1;25:e43678.
91. Willcocks C, Joy DL, Seward J, Mills S, Heywood M, Price C. Patient experiences of remote care in a pain service during a pandemic. British Journal of Pain. 2023 Feb;17(1):36-45.
92. Wilson M, Finlay M, Orr M, Barbosa-Leiker C, Sherazi N, Roberts ML, Layton M, Roll JM. Engagement in online pain self-management improves pain in adults on medication-assisted behavioral treatment for opioid use disorders. Addictive Behaviors. 2018 Nov 1;86:130-7.
93. Warmington K, Flewelling C, Kennedy CA, Shupak R, Papachristos A, Jones C, Linton D, Beaton DE, Lineker S. Telemedicine delivery of patient education in remote Ontario communities: feasibility of an Advanced Clinician Practitioner in Arthritis Care (ACPAC)-led inflammatory arthritis education program. Open Access Rheumatology: Research and Reviews. 2017 Feb 24:11-9.
94. Wilson M, Dolor RJ, Lewis D, Regan SL, Vonder Meulen MB, Winhusen TJ. Opioid dose and pain effects of an online pain self-management program to augment usual care in adults with chronic pain: a multisite randomized clinical trial. Pain. 2023 Apr 1;164(4):877-85.
95. Wilson M, Roll JM, Corbett C, Barbosa-Leiker C. Empowering patients with persistent pain using an internet-based self-management program. Pain Management Nursing. 2015 Aug 1;16(4):503-14.
96. Waddell G, O'Connor M, Boorman S, Torsney B. Working Backs Scotland: a public and professional health education campaign for back pain. Spine. 2007 Sep 1;32(19):2139-43.
97. Wilson M, Dolor RJ, Lewis D, Regan SL, Vonder Meulen MB, Winhusen TJ. Opioid dose and pain effects of an online pain self-management program to augment usual care in adults with chronic pain: a multisite randomized clinical trial. Pain. 2023 Apr 1;164(4):877-85.
98. Yilmaz E, Polat Ü. Online Disease Management Training for Older Adults With Osteoarthritis: A Randomized Controlled Trial. Musculoskeletal Care. 2024 Sep;22(3):e1931.
99. Zhao S, Chen H. Effectiveness of health education by telephone follow-up on self-efficacy among discharged patients with rheumatoid arthritis: A randomised control trial. Journal of Clinical Nursing. 2019 Nov;28(21-22):3840-7.
100. Zufferey MC, Schulz PJ. Self-management of chronic low back pain: an exploration of the impact of a patient-centered website. Patient education and counseling. 2009 Oct 1;77(1):27-32.
